# Anesthetic Management of Large Bronchogenic Cyst With Severe Tracheal Compression in Adults: A Case Report

**DOI:** 10.7759/cureus.62621

**Published:** 2024-06-18

**Authors:** Gade Sandeep, Jitendra V Kalbande, Anil Gupta, Subrata K Singha, Narendra Bodhey

**Affiliations:** 1 Anesthesiology and Critical Care, All India Institute of Medical Sciences, Raipur, Raipur, IND; 2 Anesthesiology, All India Institute of Medical Sciences, Raipur, Chhattisgarh, IND; 3 Radiodiagnosis, All India Institute of Medical Sciences, Raipur, Raipur, IND

**Keywords:** anticipated difficult airway, one lung ventilation, ez bronchial blocker, cardiopulmonary bypass, awake fiberoptic intubation, severe tracheal compression, mediastinal mass, bronchogenic cyst

## Abstract

Bronchogenic cysts (BCs) are a congenital anomaly, forming fluid-filled sacs in the bronchial tree during fetal development, and are relatively rare in adults. Patients with large BCs in the mediastinum presenting with severe tracheal compression pose a significant challenge to anesthesiologists. The confined and narrow space of the mediastinum exacerbates the compression effect on surrounding structures, leading to potential respiratory or cardiovascular collapse during anesthesia and postoperatively. Herein, we report the stepwise anesthetic management of a patient with a BC in the paratracheal region of superior mediastinum, causing near-complete tracheal compression, scheduled for right posterolateral thoracotomy and tumor excision. The patient presented with dyspnea, chest pain, cough, and severe tracheal compression necessitating meticulous airway management. Utilizing awake fiberoptic intubation with a single-lumen endotracheal tube and one-lung ventilation facilitated by an EZ bronchial blocker, we successfully secured the airway, provided ideal surgical conditions through lung deflation, and ensured perioperative safety. This case underscores the crucial role of comprehending the underlying pathophysiology, anticipating complications, and meticulously planning, preparing, and executing strategies for airway management and perioperative care in patients with mediastinal masses leading to significant tracheal compression.

## Introduction

Bronchogenic cysts (BCs) are a congenital anomaly, forming fluid-filled sacs in the bronchial tree during fetal development, and are relatively rare in adults [1]. The prevalence of BCs in the general population is unknown, and BCs account for 10-15% of mediastinal tumors and 50-60% of all mediastinal cysts [2]. Mediastinal BCs are classified into five types based on their location: paratracheal, carinal, paraesophageal, hilar, and miscellaneous [3]. While typically benign, they can lead to cardiorespiratory symptoms if they are large enough to compress the lungs or airway, necessitating surgical excision. However, the presence of large cysts presents significant challenges to anesthesiologists due to the potential for extraluminal compression and tracheal narrowing, which could lead to catastrophic outcomes without adequate planning [4].

Despite the rarity of BCs in adults, cases resulting in substantial airway compromise and necessitating surgical intervention are even less frequently documented. Moreover, there is limited literature available that discusses safe anesthesia techniques for managing such cases and their effects on patient outcomes. This report presents a detailed description of the safe, perioperative anesthetic management for excising a large BC, causing severe tracheal compression. It highlights the step-by-step approach, focusing on awake fiber-optic intubation with a single-lumen endotracheal tube (ET) and one-lung ventilation facilitated by an EZ bronchial blocker.

## Case presentation

**Figure 1:**
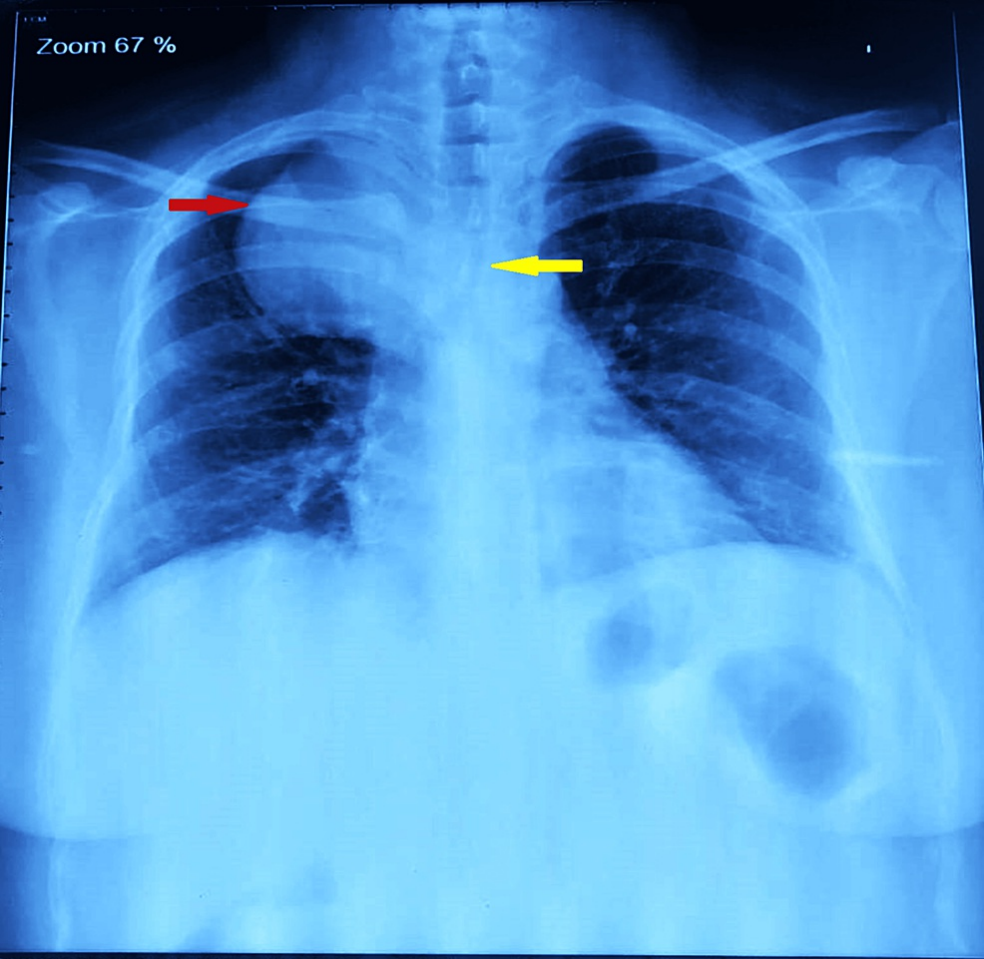
Chest X-ray PA view. The red arrow points toward the paratracheal BC. The yellow arrow points toward severe tracheal compression caused by a BC. BC, bronchogenic cyst

A 38-year-old female (weight: 79.3 kg, height: 155.8 cm, and BMI: 32.7 kg/m^2^) presented to the pulmonary department with a five-year history of dyspnea, cough, and right-sided chest pain, worsening over the past 15 days. Symptoms were partially relieved by lying on her right side. She had well-controlled diabetes mellitus (HbA1C 6.4%) with oral hypoglycemic drugs. Examination revealed reduced breath sounds on the right side with occasional rhonchi, and her oxygen saturation was 94-95%. A chest X-ray showed a rounded homogenous opacity in the right upper lobe extending into the mediastinum, with tracheal compression above the carina (Figure 1).

**Figure 2:**
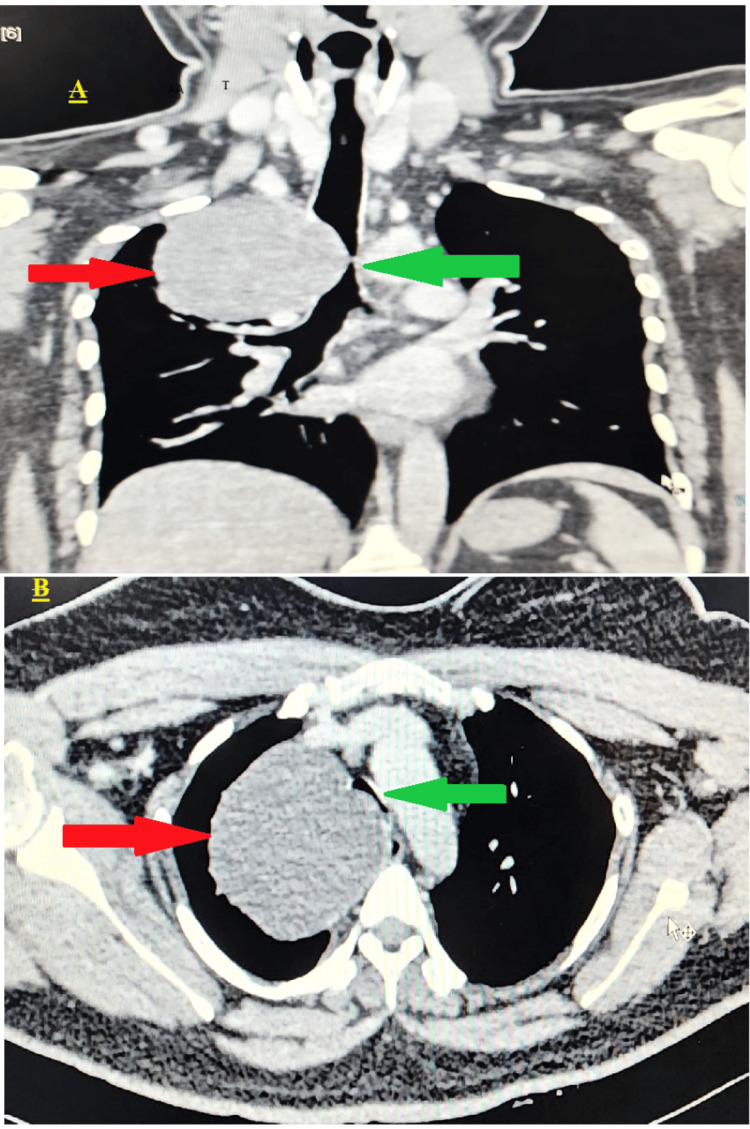
CT thorax. (A) Coronal section (red arrow showing large BC in the paratracheal region of the superior mediastinum and green arrow pointing toward severe tracheal compression of the lower one-third of the trachea). (B) Axial section (red arrow showing BC and green arrow showing severe tracheal narrowing). BC, bronchogenic cyst

CT imaging showed a solitary, well-defined, smooth, round, non-enhancing hypodense lesion measuring 7.9 × 7.3 × 6.9 cm. It was located in the medial aspect of the right upper lung zone, closely related to the right lower one-third of the trachea, and originated from the anterior to superior mediastinum. The tracheal transverse dimension, at the site of maximum compression, was 2.9 mm. The upper and lower one-third of the trachea were narrowed due to compression by the mass, which also compressed the upper one-third of the esophagus (Figure 2).

A bronchoscopy was performed to assess the tracheal narrowing further, revealing partial narrowing in the upper one-third and >90% narrowing in the lower one-third due to external compression by a cyst just above the carina level. The cyst caused dynamic airway obstruction, collapsing the trachea during expiration while allowing a small slit-like opening for breathing during inspiration. The carina was visible just 2 cm beyond the compression site (Video 1).

**Video 1:** Bronchoscopy procedure

Subsequently, the patient was referred to the cardiovascular thoracic surgery team for further management, with a provisional diagnosis of a BC. The planned procedure involved cyst excision under single lung ventilation via right posterolateral thoracotomy. Hematological and biochemical investigations were within normal limits.

A thorough preanesthetic assessment was conducted, and with a Mallampati airway score of 3, the patient was accepted for surgery with an American Society of Anesthesiologists physical status (ASA-PS) classification II. The anesthesia plan included awake fiber-optic intubation using a single-lumen ET, followed by general anesthesia and one-lung ventilation with an EZ bronchial blocker, along with thoracic epidural analgesia. Before surgery, the patient was encouraged to perform incentive spirometry and chest physiotherapy. Nebulization with budecort twice daily and duolin every eight hours, along with antibiotics (ceftriaxone 1 gm BD), was initiated. Routine premedication, with care to avoid sedatives, was administered according to institutional protocol. The patient received education and counseling regarding the awake fiberoptic procedure, analgesic technique, and anesthetic plan.

On the day of the surgery, in the preoperative area, the patient was nebulized with 4 mL of 4% topical lignocaine for 15 minutes. Moreover, 0.1% xylometazoline drops were instilled in each nostril (two drops in each nostril). Before shifting the patient to the operating theater (OT), a difficult airway cart, including a fiberoptic bronchoscope, was prepared. The patient was then wheeled into the OT, and ASA standard monitors were attached. Under local anesthesia, a 16G IV cannula was secured in the right hand, and the left radial artery was cannulated for invasive blood pressure (BP) monitoring. In a sitting position, an epidural catheter was aseptically inserted at the T5-T6 interspace and secured 9 cm from the skin. Subsequently, in a supine position, oxygen was administered via nasal prongs at a rate of 4 liters per minute. A dexmedetomidine infusion at a rate of 0.5 mcg/kg/min was initiated. A landmark-guided bilateral superior laryngeal nerve block was performed using 2% lignocaine (2 mL injected on each side). A transtracheal airway block was administered with 4% topical lignocaine (2 mL injected after confirming the needle placement by aspiration of an air bubble). The cardiopulmonary bypass (CPB) machine was kept on standby, and femoral vessels were painted, draped, and kept exposed.

After achieving sufficient sensory anesthesia under spontaneous respiration, awake fiberoptic bronchoscopy was performed using a 3.1 mm outer diameter pediatric bronchoscope (Olympus LF-DP, Tokyo, Japan). The bronchoscopy confirmed the complete collapse of the lower one-third of the trachea during expiration, with a small slit-like gap observed during inspiration. Through this gap, the tip of the bronchoscope was carefully negotiated and positioned distal to the lower one-third of the tracheal narrowing. The operating table was tilted to the right lateral position, and the patient was instructed to hold her breath during inspiration. Keeping the carina under vision, a single-lumen, 7-mm internal diameter cuffed ET was rail-loaded over the bronchoscope. The tip of the ET tube was positioned just distal to the narrowing, as confirmed using capnography tracing and bronchoscopy. General anesthesia was induced using titrated doses of intravenous fentanyl and propofol until loss of consciousness and apnea were achieved. Following confirmation of the possibility of manual assisted ventilation, succinylcholine 150 mg was administered. Bilateral breath sounds were rechecked, and even chest expansion was observed. Post-induction, oxygen saturation was 99%, BP measured 108/68 mmHg, and peak airway pressure was 20 mmHg. The tube was secured at 20 cm and positioned in the right corner of the mouth.

**Figure 3:**
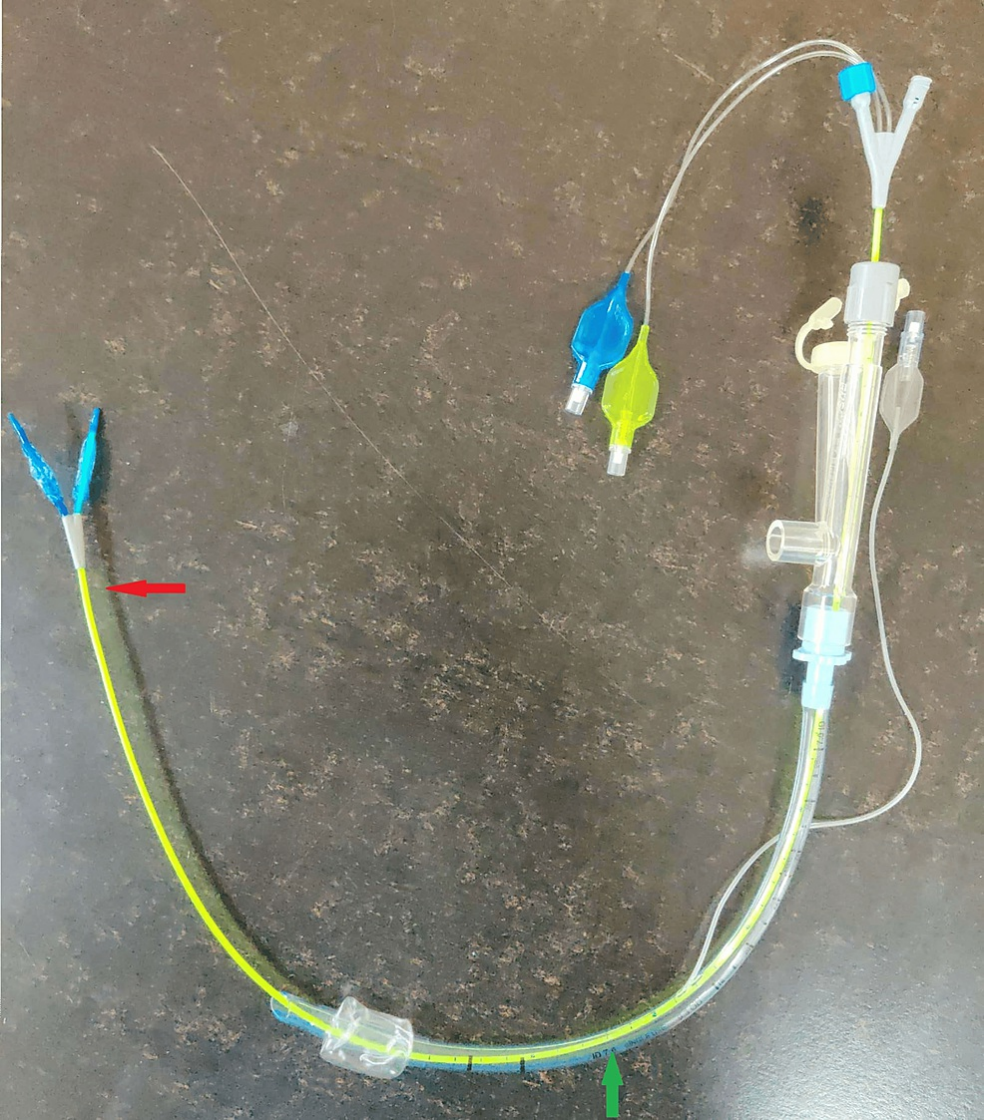
EZ bronchial blocker. The green arrow points to the ET, and the red arrow points to the Y-shaped tip of the blocker. ET, endotracheal tube

A multiport adapter was connected to the ET tube. The bronchoscope was reinserted through the multiport adapter, and the distance of the tip of the ET tube from the carina was 2 cm. A Rush EZ bronchial blocker (Teleflex Medical Dublin Road, Athlone, Ireland), 7 French, 75 cm, was inserted under the guidance of a bronchoscope along the third port of the adapter (Figure 3).

Initially, both bronchial cuffs were deflated and advanced toward the carina, but twice, they both inadvertently entered the right bronchus.

**Figure 4:**
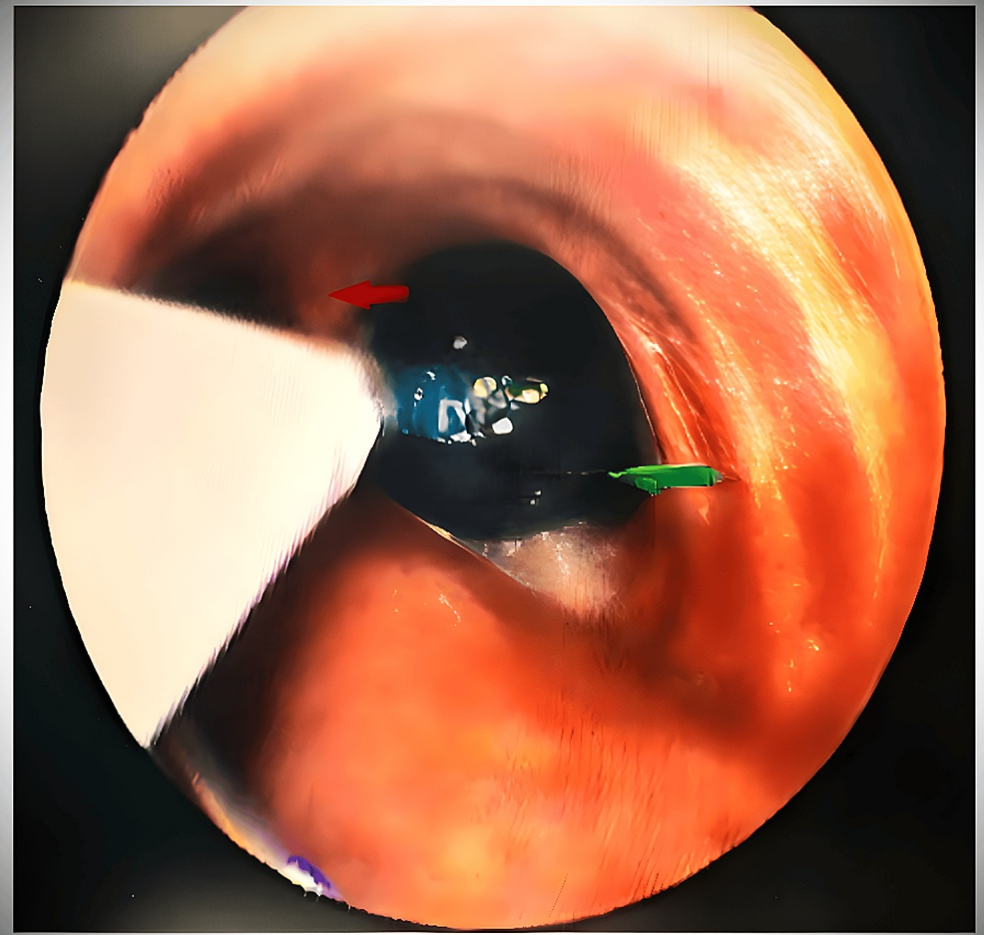
Y-shaped tip of EZ bronchial blocker, stranded on carina. The red arrow points to the carina, and the green arrow points to the inflated cuff of the right arm of the bronchial blocker inside the right bronchus.

We withdrew the EZ blocker, and adjustments were made by repositioning the ET tube from the right corner of the mouth to the center. Then again, the EZ blocker was advanced until the Y-shaped distal end was stranded on the carina, confirming that one extension had been positioned in each bronchus. The right bronchial cuff was inflated with air, and the minimal occlusion volume (10 mL) to obliterate the lumen was noted under bronchoscopic visualization (Figure 4).

With auscultation, lung isolation was confirmed. Volume-controlled ventilation was initiated for two-lung ventilation, and the patient was then placed in the left lateral decubitus position for the right posterolateral thoracotomy. The position of the bronchial blocker was reconfirmed with fiberoptic bronchoscopy and auscultation. Single lung ventilation was instituted upon the surgeon’s request by inflating the right lumen of the bronchial blocker. Pressure-controlled ventilation began for single-lung ventilation targeting peak airway pressures of less than 35 cmH20, plateau pressures of less than 25 cmH20, and positive end-expiratory pressure (PEEP) of 5-10 cmH20. Anesthesia depth was maintained with a combination of inhalational agents (50% oxygen, 50% air, and isoflurane 1% with a MAC age of 0.5-0.6), muscle relaxant (injection vecuronium), and epidural infusion (0.25% injection Bupivacaine with 2 μg/ml of fentanyl) at 4 mL per hour, adjusted according to BP. Continuous monitoring included ECG, SPO2, ETCO2, airway pressure, and invasive BP to detect any hypoxemia, desaturation, or arrhythmias.

**Figure 5:**
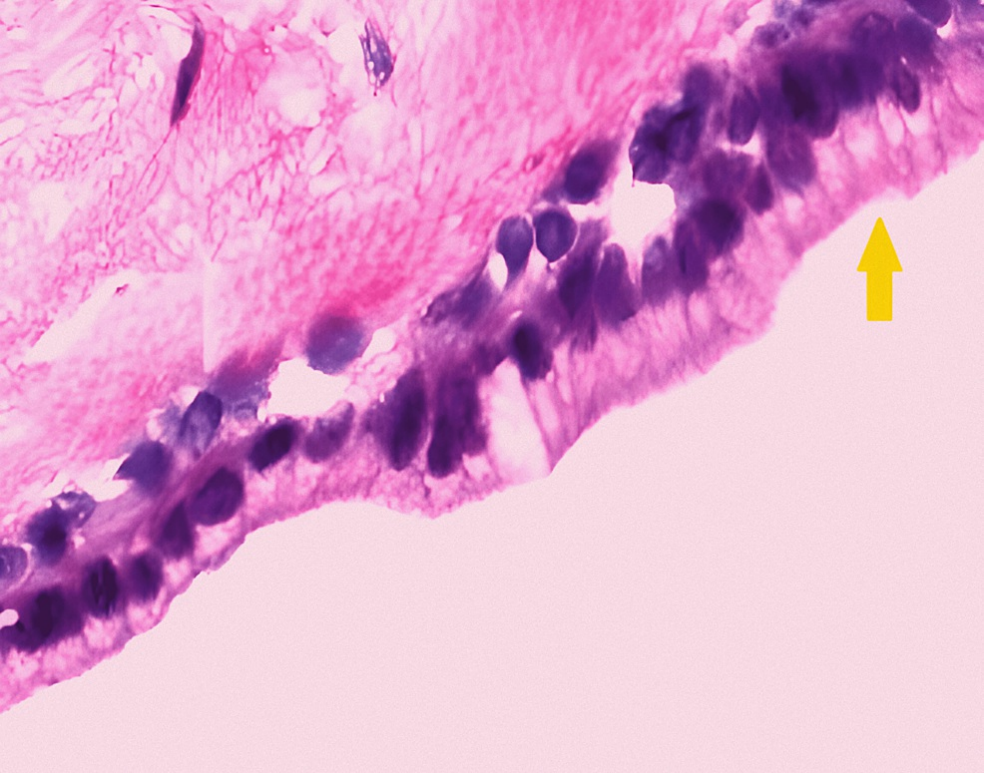
Histopathology image of the bronchial cyst (yellow arrow showing columnar epithelium)

Surgical findings revealed a large, rounded BC measuring around 8 × 7 × 8 cm in size in the right upper lobe posteriorly adhered to the trachea and esophagus, inferiorly to the azygous, and superiorly to the superior vena cava (SVC). The cyst was dissected, 40 ml of whitish-thick fluid was drained out, and the cyst wall was excised. The intraoperative course was uneventful. Following cyst removal, two-lung ventilation was resumed, and neuromuscular blockade was reversed using an injection of neostigmine 3 mg and an injection of glycopyrrolate 0.6 mg, and the patient was extubated on the operating table. Histological examination revealed a ciliated columnar lining, which confirmed that the lesion was BC (Figure 5).

**Figure 6:**
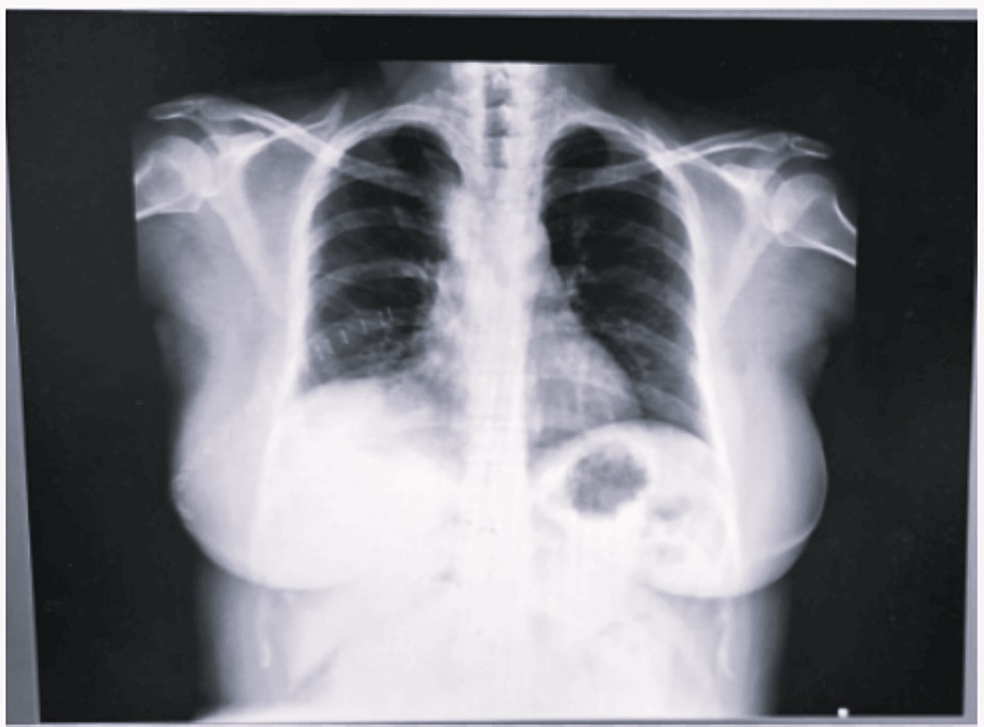
Postoperative chest X-ray PA view

Postoperatively, the patient was observed in the ICU, during which analgesia was provided with a continuous epidural infusion (0.125% injection bupivacaine with 2 μg/ml injection fentanyl) of 3-4 ml/hr and an injection of paracetamol (1 gm, eighth hourly) was administered. Incentive spirometry and chest physiotherapy were advised to improve the respiratory mechanics of the patient (Figure 6). The postoperative course was uneventful, and the patient was discharged on the seventh day postoperatively. The patient was followed up for one year, with no recurrence of the BC.

## Discussion

BCs, congenital lesions found in the mediastinum or lungs, occur mostly in infants and rarely in adults. They are foregut-derived cystic malformations of the respiratory tract, typically originating in the middle mediastinum (65-90%), including the right paratracheal area (20%) and the tracheal carina (50%) [1,3]. Surgical excision is usually required for large cysts causing compressive symptoms, with various approaches available such as sternotomy, thoracotomy, cervical mediastinoscopy, anterior mediastinoscopy, and video-assisted thoracoscopic surgery.

A literature search revealed very few cases of BCs in adults, presented as mediastinal masses causing severe tracheal compression. Patients with large BC in the mediastinum present numerous significant anesthesiological challenges, with one of the most critical being the potential occurrence of mediastinal mass syndrome (MMS) [5]. In our case, as the cyst caused more than 90% tracheal compression, this translated into concerns about managing a difficult airway due to severe tracheal narrowing, performing awake fiberoptic bronchoscopy while maintaining spontaneous ventilation (SV), securing the airway without causing trauma or cyst rupture, administering general anesthesia in the compromised airway, preparing for standby CPB, and addressing the possibility of MMS leading to desaturation, airway collapse, respiratory failure, arrhythmias, and hemodynamic collapse. Other considerations included lateral positioning, lung isolation with an EZ bronchial blocker, and managing complications related to one-lung ventilation such as hypoxemia and desaturation.

A thorough preoperative assessment is essential for patients with mediastinal masses. Anesthesiologists evaluate clinical, functional, bronchoscopic, and radiological data to assess risks, focusing on tumor anatomy and its relationship with surrounding structures. Symptoms indicating airway compromise (cyanosis, stridor, and dyspnea) and cardiovascular issues (palpitation and SVC syndrome) are crucial. Evaluating these symptoms while patients are awake in various positions, especially supine and lateral decubitus, helps identify a comfortable “rescue position,” optimizing respiratory and hemodynamic function.

Preoperatively, various tests, including X-rays, CT scans, MRI, ECG, ECHO, and PFTs, offer detailed anatomical information about masses, their impact, and their relationships with surrounding structures. These tests aid in preoperative risk assessments, formulating a deliberate plan for both anesthetic and surgical management. Bronchoscopy plays a critical role in preoperative assessment and intraoperative guidance by visualizing the trachea, proximal airways, and segmental airways to evaluate potential extrinsic airway compression from large mediastinal masses [6]. Assessing position preferences and the severity of airway compression in different positions aids in anesthesia and surgical planning.

General anesthesia reduces lung volume and functional residual capacity, relaxes smooth airway muscles, and increases large airway compressibility. It also flattens the trachea through extrinsic compression and muscular relaxation and disturbs active airway inspiratory force. Factors such as the supine position, endotracheal intubation, eliminating glottic regulation, the size and location of the mediastinal mass, and preexisting airway diseases can contribute to airway collapse. Therefore, avoidance of general anesthesia, particularly paralytic agents, or maintenance of SV is recommended [7]. Stepwise induction should be followed, avoiding deep sedation. While no single agent is considered superior, any agent should be used cautiously, considering the preservation of SV.

For those with central airway obstruction, the safest approach is to secure a distal airway via awake fiberoptic intubation, positioning the ET beyond the tracheal obstruction [8]. This method permits spontaneous breathing without the influence of general anesthesia, enables assessment of tracheal compression level and degree, is reversible, and can be aborted at any point. Since our patient had positional dyspnea and significant tracheal compression (>90%), there was an increased risk of perioperative airway decompensation. Therefore, awake fiberoptic intubation was planned in our patient; the airway was anesthetized with lignocaine nebulization and blocks, and sedation was administered with dexmedetomidine at a dose of 0.5 µg/kg/h. Dexmedetomidine (dose range of 0.2-0.7 µg/kg/h) has been shown to help maintain SV and reduce the risk of complete airway obstruction in the management of mediastinal masses [9]. During the procedure, as our patient was spontaneously breathing with dynamic tracheal obstruction, we instructed her to hold her breath during inspiration and slightly tilt the operating table to the right. This approach aimed to maximize the tracheal diameter, facilitating the smooth passage of the ET into the trachea without risking airway trauma or cyst rupture. Post-intubation ventilation depends on the placement of the tube relative to the tracheobronchial obstruction. If the tube is distal to the obstruction, manual ventilation can gradually be initiated, and if tolerated well, positive pressure ventilation with or without muscle relaxants can be employed [8]. We used succinylcholine after intubation, followed by vecuronium for maintenance after confirming successful positive pressure ventilation.

Lung isolation with a double-lumen tube (DLT) is preferred due to its higher safety margin compared to a bronchial blocker [10]. However, disadvantages include being more rigid, potential injuries to the larynx and trachea, difficulties in abnormal airway anatomy, malposition, the need for bronchoscopy for positioning, a higher incidence of laryngoscopy reattempts during intubation, and an increased cardiovascular response during intubation. In our case, due to the partial narrowing of the tracheal lumen in the upper one-third and complete narrowing in the lower one-third, insertion of a DLT could have been difficult and might have caused trauma to the airway or rupture of the cyst. Bronchial blockers are an alternative to DLTs for lung isolation. Despite the concerns of inadequate lung isolation and migration of the bronchial blockers, they do provide a choice in cases where DLT is contraindicated or insertion is deemed difficult [11]. Likewise, we preferred the use of a single-lumen ET with a bronchial blocker (EZ blocker) for lung isolation over the DLT. The EZ blocker is a semirigid Y-shaped bronchial blocker with two distal extensions, either of which can be inflated to block the required bronchus and reduce the chances of its malposition compared to other blockers [12]. The position of this bronchial blocker must be confirmed by a fiberoptic bronchoscope. The insertion of an EZ blocker requires at least a 7-mm internal diameter single-lumen tube and a multiport adapter. Properly deploying the Y-shaped distal part of the EZ blocker requires a minimum of 4 cm distance between the distal end of the tube and the carina, which could be a matter of concern in Indian females due to their mean (SD) distance between the tip of the tube and the carina being 2.28 (1.55) cm, significantly less than the required cutoff of 4 cm [13,14]. In our case, the distance between the tip of the ET tube and the carina was 2 cm, so we were unable to deploy the Y-shaped distal part into the carina on the first two attempts. However, shifting the proximal part of the ET tube from the corner to the center of the mouth facilitated successful deployment.

Single-lung ventilation can lead to hypoxemia and desaturation. Adequate measures (continuous positive airway pressure for non-ventilated lungs and PEEP for ventilated lungs, etc.) must be taken to prevent desaturation during single-lung ventilation [15]. The close proximity of the cyst to the great vessels and the heart makes the patient prone to developing arrhythmias and hemodynamic instability. As the cyst in our patient was present from the anterior to the superior mediastinum, continuous invasive monitoring of the SPO2, ETCO2, serial ABG, invasive BP, and electrocardiogram was important. The necessity of the insertion of a central venous catheter and the use of transesophageal echocardiography depends on the patient’s comorbidities and the nature of the mediastinal mass.

Inadequate analgesia in thoracotomy cases is associated with a negative outcome in terms of patient recovery. Multiple sensory afferents supply the nociceptive signals following thoracotomy. Multimodal analgesic techniques have to be employed for adequate intraoperative and postoperative analgesia [7]. We used thoracic epidural, intravenous fentanyl, skin infiltration, and paracetamol to provide perioperative analgesia.

CPB may be indicated when ventilation is compromised after induction, such as in cases of tumor compression affecting the distal third of the trachea, both mainstem bronchi, and the carina. It is also warranted in situations of extensive tumor compression of the right heart or pulmonary artery, posing a significant risk of hemodynamic collapse [7,8]. These events can occur unexpectedly at any stage of anesthesia, including induction, positioning, surgery, emergence, extubation, or postoperatively in the PACU. In our case, CPB was kept on standby with exposed femoral vessels preoperatively. Tempe et al. [16] cannulated the femoral vessels and kept CPB ready because it was thought that there was a definite danger of the patient developing airway obstruction. Following surgery, patients classified as unsafe and high risk should be transferred and monitored in the ICU [3]. It is important to recognize that the completion of the operation does not eliminate the need for vigilance. Postoperatively, we also monitored the patient in the ICU.

The literature review revealed a few case reports that have described different approaches to managing BCs located in various mediastinal regions. For instance, Sulen et al. [17] employed awake fiberoptic intubation with an endobronchial tube for a cyst in the posterior mediastinum, while Wools et al. [18] conducted an emergency tracheostomy for cysts in the superior mediastinum, which presented with a stridor and was later managed with a thoracotomy. Furugen et al. [19] addressed a recurrent BC tightly attached to the left atrium through sternotomy and CPB. Additionally, Thakur et al. [20] described a BC in a four-year-old female child located in the middle mediastinum, extending to the subcarinal region, managed with intravenous ketamine and inhalational induction with endotracheal intubation. In our case, a cyst in the superior mediastinum causing severe tracheal compression was effectively managed with awake fiberoptic intubation using a single-lumen tube and an EZ bronchial blocker without the need for CPB. Therefore, our case underscores the importance of adapting management strategies to the unique characteristics and clinical presentation, particularly when encountering severe tracheal compression in the superior mediastinum.

## Conclusions

Effective management of large BCs causing severe tracheal compression in adults demands a thorough preoperative evaluation, a comprehensive understanding of the pathophysiology, meticulous planning, and precise execution of perioperative management strategies. By employing awake fiberoptic intubation using a single-lumen ET, one-lung ventilation using an EZ bronchial blocker, and multimodal analgesia, we effectively secured the airway, ensured optimal surgical conditions, and provided adequate pain control for safe tumor excision. Continuous monitoring and preparedness for CPB were also maintained to address any hemodynamic instability or respiratory compromise swiftly. Collaborative interdisciplinary care, continuous monitoring, and prompt adaptation to intraoperative challenges are essential for optimizing outcomes in these challenging cases. By adhering to evidence-based practices and maintaining a high level of clinical expertise, favorable outcomes can be achieved for these complex surgical cases.
